# Inhibition of Chondrosarcoma Growth by mTOR Inhibitor in an In Vivo Syngeneic Rat Model

**DOI:** 10.1371/journal.pone.0032458

**Published:** 2012-06-27

**Authors:** Jennifer Perez, Anne Valérie Decouvelaere, Thomas Pointecouteau, Daniel Pissaloux, Jean Philippe Michot, Anthony Besse, Jean Yves Blay, Aurélie Dutour

**Affiliations:** 1 Department of Clinical Sciences, Centre de Recherche en Cancérologie UMR Inserm U1052-Equipe 11- CLB – Lyon, France; 2 Department of Biopathology and Cytology, Centre Léon Bérard, Lyon, France; 3 Department of Medical Oncology, Centre Léon Bérard, Department of Clinical Sciences, Centre de Recherche en Cancérologie, Lyon, University Claude Bernard Lyon 1, Lyon, France; 4 Department of Clinical Sciences, Centre de Recherche en Cancérologie UMR Inserm U1052-Equipe 11- CLB – Lyon, France; University of Chicago, United States of America

## Abstract

**Background:**

Chondrosarcomas are the second most frequent primary malignant type of bone tumor. No effective systemic treatment has been identified in advanced or adjuvant phases for chondrosarcoma. The aim of the present study was to determine the antitumor effects of doxorubicin and everolimus, an mTOR inhibitor on chondrosarcoma progression.

**Methods and Findings:**

Doxorubin and/or everolimus were tested *in vivo* as single agent or in combination in the rat orthotopic Schwarm chondrosarcoma model, in macroscopic phase, as well as with microscopic residual disease. Response to everolimus and/or doxorubicin was evaluated using chondrosarcoma volume evolution (MRI). Histological response was evaluated with % of tumor necrosis, tumor proliferation index, metabolism quantification analysis between the treated and control groups. Statistical analyses were performed using chi square, Fishers exact test. Doxorubicin single agent has no effect of tumor growth as compared to no treatment; conversely, everolimus single agent significantly inhibited tumor progression in macroscopic tumors with no synergistic additive effect with doxorubicin. Everolimus inhibited chondrosarcoma proliferation as evaluated by Ki67 expression did not induce the apoptosis of tumor cells; everolimus reduced Glut1 and 4EBP1 expression. Importantly when given in rats with microscopic residual diseases, in a pseudo neoadjuvant setting, following R1 resection of the implanted tumor, everolimus significantly delayed or prevented tumor recurrence.

**Conclusions:**

MTOR inhibitor everolimus blocks cell proliferation, Glut1 expression and HIF1a expression, and prevents *in vivo* chondrosarcoma tumor progression in both macroscopic and in adjuvant phase post R1 resection. Taken together, our preclinical data indicate that mTOR inhibitor may be effective as a single agent in treating chondrosarcoma patients. A clinical trial evaluating mTOr inhibitor as neo-adjuvant and adjuvant therapy in chondrosarcoma patients is being constructed.

## Introduction

Chondrosarcomas constitute a heterogeneous group of neoplasms accounting for 20% of bone malignancies, that have in common the production of cartilage-like matrix by the tumor cells [1]. Clinical management of these second most common type of skeletal malignancies after osteosarcoma has remained largely unchanged over the last 3 decades [2]. Because of their extracellular matrix, low percentage of dividing cells, and poor vascularity, chondrogenic tumors are relatively chemo- and radiotherapy resistant [2], [3]. Chemotherapy and radiation have not been tested for efficacy, but in clinical routine they are not considered as active for the treatment of this disease and surgery still prevails as the primary treatment modality of this tumor [2], [3]. The 10-year survival rate of chondrosarcoma being unchanged over the past 40 years and ranging from 29–83% [1], [4] depending on the chondrosarcoma subtype and grade. Improving chondrosarcoma clinical management is therefore a challenging problem and novel therapeutic approaches are needed.

The idea of targeting mTOR as anticancer strategy emerged less than a decade ago and became rapidly a focus for cancer therapeutic developments [5], [6]. MTOR is a ubiquitously expressed serine/threonine kinase that affects a number of cellular functions, from protein synthesis to cell proliferation. MTOR is also a point of convergence in many signalling pathways that respond to growth factors and stress/energetic status [7], [8]. MTOR integrates all these signals and acts by modulating the phosphorylation of p70S6 kinase (p70S6K/S6K1) and 4E binding protein 1 (4E-BP1) leading to protein synthesis and cell cycle progression (G1 to S phase transition) [9]. MTOR is a central regulator in cellular processes (metabolism, survival, proliferation) upon which tumor cells depend and there are growing data indicating that many cancers present alteration upstream and downstream of mTOR leading to this pathway abnormal activation [5], [10]. Thus mTOR represents a potential therapeutic target and efforts have been made to develop inhibitors specific for this protein [6], [11].

Rapamycin (sirolimus) and its analogues temsirolimus and everolimus have shown specific mTOR inhibition and anticancer activities in preclinical trials [12]–[14]. Previous studies have shown that specific mTOR inhibitor used as monotherapy or in combination with other agents had an antitumoral effect in solid or haematological malignancies [15], [16]. Pivotal clinical trials with mTOR inhibitors are ongoing in solid tumors including neuroendocrine tumors, breast cancer, gastric cancer [6]. Recently a case report of a response to an association of rapamycin and cyclophosphamide in a case of myxoid chondrosarcoma was published pointing out a possible role of this approach in clinical setting [17].

Based on these data and on studies showing additive effects of mTOR inhibitor with chemotherapy [14], [15], [18], [19], the antitumor effect of a combination of chemotherapy and/or everolimus, an mTOR inhibitor was tested in a preclinical rat chondrosarcoma model. We present here the results of this study.

## Methods

Care of and procedures for animals were performed according to institutional and national guidelines. The study was approved by the Cermep ethics committee (Cermep COMEX) and registered under the ID: DUTOUR_Chondro01/03. Animals were housed and experiments were carried out at Cermep a structure approved for housing and small animal experimentations (agreement number: A 69 383 05 01). For each tumor model, three experiments were carried out.

Animals were anesthetized throughout all surgical and imaging procedures with isoflurane/oxygen (2.5%/2.5%, v/v) (Minerve, Esternay, France).

Rat chondrosarcoma model.

### Primary Chondrosarcoma Model

The transplantable orthotopic rat chondrosarcoma has been described [20]. This model is a grade II chondrosarcoma with mild cellular atypia that mimics its human counterpart in terms of aggressiveness and chemoresistance phenotype. Tumors were grafted on 25-days-old Sprague–Dawley rats (Charles River Laboratories, L’Arbresle, France). Briefly, tumor fragments (10 mm^3^) were transplanted on the right posterior tibia of the rats after periostal abrasion. At day 12 after tumor transplantation, animals underwent a first MRI and were randomly divided into the following groups: i) Control (saline; n = 7); ii) doxorubicin (1 mg/kg; n = 7) (Doxorubicin; Baxter, Deerfield IL, USA); iii) everolimus (1 mg/kg; n = 7) (Certican®, Novartis; Rueil-Malmaison, France); iv) everolimus + doxorubicin (1 mg/kg each; n = 7). Doxorubicin is an agent commonly used in the treatment of musculoskeletal sarcoma and was therefore chosen as “reference treatment” in our study. Treatment was administered IP twice a week starting day 12 and for 3 weeks, animals were imaged every 10 days throughout treatment. Previous studies conducted in our group showed that the dose of 1 mg/kg of doxorubicin and everolimus is well tolerated and effective in the rat chondrosarcoma model. Increasing the doses (for each compound) accrued little antitumor activity. Thus 1 mg/kg of everolimus and doxorubicin appeared to be the optimal dose in our sarcoma model. All animals were euthanized if tumor were too bulky or if any signs of distress were observed.

### Model of Local Tumor Recurrence

Primary chondrosarcoma were obtained as described in the previous paragraph. When the tumors reached a volume of approximately 500 mm^3^ (and considered progressive tumors), the animals underwent an intralesional curettage [20]. One day after intralesional surgery, treatment was initiated. Rats were treated with everolimus alone at the dose of 1 mg/kg twice a week, or with doxorubicin alone (1 mg/kg) twice a week or by saline (as vehicle) for 3 weeks or till tumors reached the size of 2 cm in the largest diameter. Rats were imaged throughout treatment by MRI. All animals were euthanized if tumor were too bulky or if any signs of distress were observed. At the conclusion of the study, tumors were dissected, weighed, and processed for further analysis.

### Tumor Growth Assessment

Two perpendicular diameters were measured using a caliper twice a week and tumor volume was estimated using the formula V = 0.5*0.5*a*b^2^, where a and b were respectively, the largest and smallest perpendicular tumor diameters.

Chondrosarcoma-bearing rats underwent MRI examinations on days 0, 10 and 20 after initiation of treatment.

MRI acquisition was performed 15 min after intravenous administration of gadolinium. MRI acquisitions were performed on a Bruker 7T Biospec system (Bruker, Ettlingen, Germany) equipped with 400 mT/m gradient set, using an emission/reception body coil (o.d. = 112 mm and i.d. = 72 mm). Then, T2-weighted contrast images were acquired in the axial and coronal plane based on a fat suppressed (FS) rapid acquisition with relaxation enhanced (RARE) sequence with the following parameters: repetition time (TR) 3625,2 ms, echo time (TE) 60 ms, RARE factor  = 8 and 3 min scan time. T1-weighted contrast images were acquired in the axial and coronal plane based on a fat suppressed (FS) spin echo (MSME) sequence with the following parameters: repetition time (TR) 584,4 ms, echo time (TE) 10,7 ms and 4 min scan time. For both sequences, a total of 25 slices (slice thickness 1 mm) was acquired with a field of view (FOV) of 7×7 cm^2^, matrix 256×192, resulting in an in-plane resolution 273×365 µm^2^.

### Western Blotting

Immunoblotting was done to confirm everolimus inhibitor activity and the upstream and downstream consequences of mTOR inhibition. Tumor samples were pulverized under liquid N_2_, and extracted as described previously. Immunoblotting procedures have been previously reported. All proteins were detected, after dosing, by resolving proteins on Criterion Mini Protean 4–15% SDS-PAGE (Bio-Rad Laboratories, Marne La Coquette, France) and blotted onto nitrocellulose membrane. The following primary antibodies were used: anti-phospho-AKT (S473; 1∶1,000), anti-phospho-S6K (1∶1,000), anti- 4EBP1 (1∶1,000) anti-phospho-mTOR (1∶1,000); anti- AKT (S473; 1∶1,000), anti- S6K (1∶1,000; Inc.), anti-mTOR (1∶1,000). All were from Cell Signaling Technology (Danvers MA, USA). Immunoreactive bands were visualized by using ECL Plus (GE Healthcare, Orsay, France) and Biomax XAR film (Kodak) after incubation with the horseradish peroxidase–conjugated secondary antibody (1∶5,000; Millipore; Molsheim; France). The ratio of phosphorylated to total signals was quantified unsing ImageJ software (http://rsbweb.nih.gov/ij/).

### Histologic Analyses

At the endpoint of study, histologic characterization and immunohistologic analyses were performed on tumors from representative animals of all groups. Tumors samples were fixed in formalin solution embedded in paraffin and cut at a thickness of 5 µm for Ki67 and Glut-1 staining, For phospho-4EBP1 and phospho-Akt staining, sections were embedded in OCT, frozen and cut at a thickness of 5–6 µm. For immunostaining the following primary antibodies were used: anti Ki-67 (1∶50 Dako, Trappes, France), anti-phospho-4EBP1 (1∶400; Cell Signaling technology), anti-phospho-Akt (1∶400; Cell Signaling technology), anti-Glut-1 (1∶100; Abcam; Cambridge, MA USA). Detection of Ki67 and Glut-1 immunostaining were performed using Vectastain ABC Kit (Vector Laboratories, Burlingame, CA USA) according to manufacturer’s instructions, followed by counterstaining using hematoxylin (Sigma-Aldrich). Phospho-Akt and phospho-4EBP1 were visualized using Texas Red-conjugated anti-mouse secondary antibody (Vector Laboratories).

For quantitative assessment of Ki67 staining, a total of 200 tumor cells were evaluated per slide (in fields showing the highest cell density (periphery of the tumor)) within an examination area of 0.196 mm^2^. Glucose transporter 1 (Glut-1) staining was graded as positive or negative. Cases were considered negative when less than 10% of cells showed Glut-1 staining and positive when 10% or more of tumor cells showed Glut-1 staining. Variations in staining intensity of the cells were scored, and the following criteria were used: +, weak but unequivocal staining in some cells; ++, staining of moderate intensity; and +++, strong or intense staining. All IHC slides were interpreted by two independent observers, one being a qualified pathologist with no knowledge of the clinicopathologic variables evaluated in the specimens.

### Quantitative Real-time PCR

Total RNA was extracted from representative tumors from all groups using Rneasy Mini Plus Kit (Qiagen, Courtaboeuf France) according to the manufacturer’s instructions. First-strand cDNAs were generated in reverse transcriptase reactions containing 1 µg total RNA and Quantitect Reverse Transcription kit (Qiagen). Gene expression of rat HIF1α, GLUT-1 and HPRT was quantified on a Applied thermocycler (Applied 7000) using QuantiFast SybrGreen PCR kit and Quantitect primers (Qiagen). For RT-PCR singleplex reactions, a final volume of 25 µL per 2.5 µL cDNA were diluted in RNase-free water,12 µL Quantifast Master Mix, and 2.5 µL of primers. Amplification conditions were set up to 5 min at 95°C followed by 40 PCR cycles (10 s at 95°C, 30 s at 60°C). The quantity of HIF1α and GLUT-1 cDNA detected in each reaction was normalized to HPRT and expressed as a ratio of sample cDNA to HPRT cDNA.

### Statistical Analysis

Data points are given as mean values ± standard deviation. Results were compared by the nonparametric Mann–Whitney U test, due to sample size. A p-value <0.05 was considered statistically significant.

## Results

### Everolimus Blocks chondrosarcoma Progression

**Figure 1:**
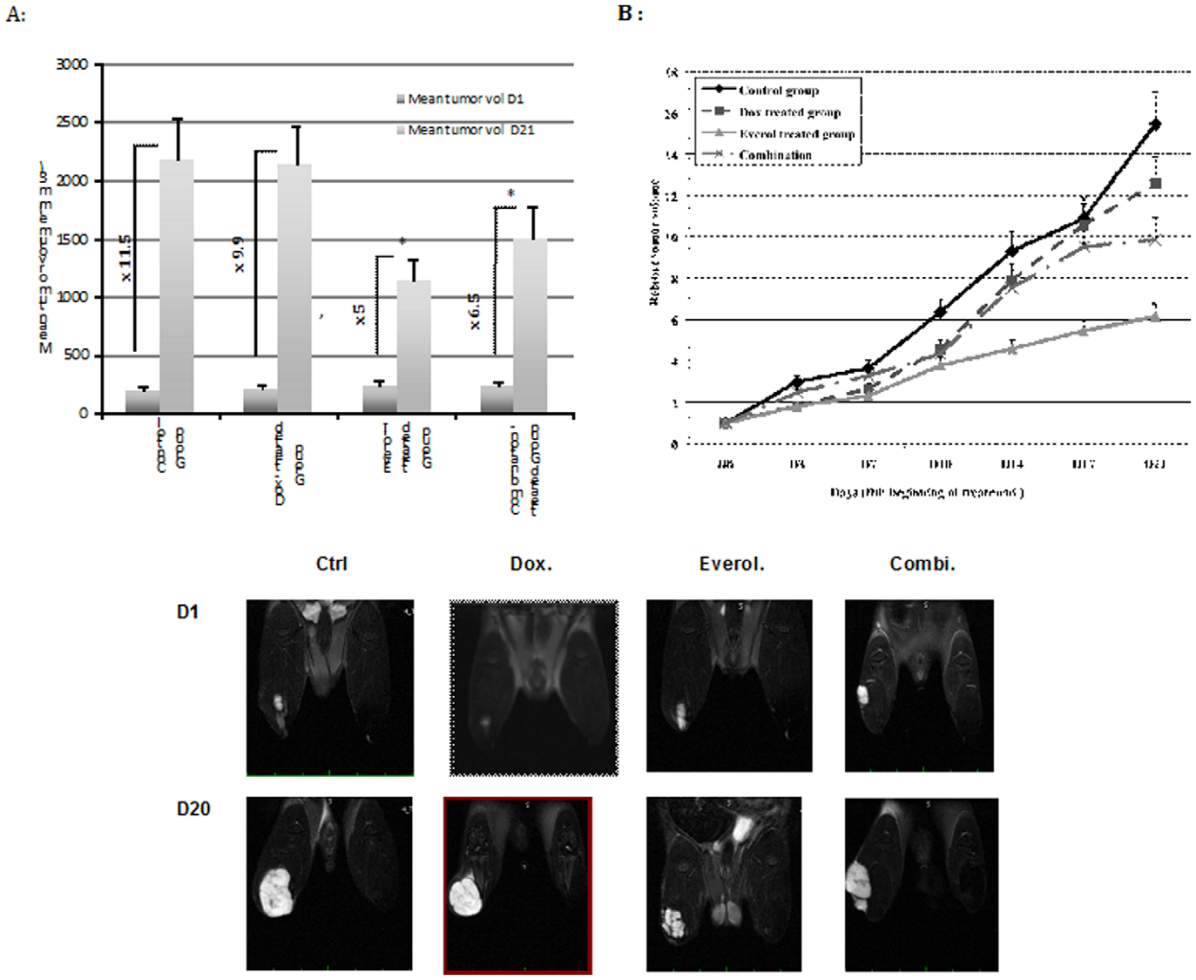
Response of established chondrosarcoma model to each treatment. A, Mean tumor volume evolution between day 1 and day 21 (fold expansion are expressed beside each group). B:Time course of mean tumor volume (expressed in fold expansion compared to day 1). The results indicated that everolimus as single agent significantly (p<0.01) slowed down chondrosarcoma progression; the combination of doxorubicin to everolimus showed an additive effect in comparison to doxorubicin alone, but was less efficient than everolimus alone. C: Longitudinal growth of tumor of one rat from each group monitored by MRI.

To determine whether the combination of everolimus and doxorubicin is therapeutically useful we examined the antitumor activity of the individual agents and the combination of everolimus with doxorubicin in the established orthotopic chondrosarcoma model (Table 1 and Figure 1A). In these setting, data presented are one experiment representative of three experiments. There was no significant differences in tumor progression and mean tumor volumes among the doxorubicin treated group and the control group: at day 21 the mean tumor volume in the doxorubicin treated group was 2130 mm3 (+/−330 mm3) and 2165 mm3 (+/−370 mm3) in the control group (p>0.05). In contrast, everolimus used as single therapy yielded an inhibition of tumor progression but with no volumetric tumor regression (Table 1 and Figure 1). Significant (p = 0.001 at day 21) differences in average tumor size were observed starting day 10 after initiation the treatment between the everolimus treated groups and the control group, and from day 14 between the everolimus and doxorubicin-treated groups (Figure 1B; p = 0.02 at day 21). Figure 1C showed a representative MRI of tumor progression in the different groups: the time to reach a relative tumor volume of 10 times the initial tumor volume (T10) was 14 days in the control group, 16 days in the doxorubicin group. Tumors in the everolimus treated group did not reach this 10-fold value (Figure 1A). Everolimus resulted in an approximately 55% inhibition of tumor growth at day 21 compared to either control or doxorubicin groups (Table 1; p<0.05).

**Table 1:** Summary statistics of tumor evolution for each treated group.

	Control Group(n = 7)	Doxorubicin-treatedGroup (n = 7)	Everolimus-treatedGroup (n = 7)	Combination (everol+doxor.) Group (n = 7)
**Mean tumor vol (mm3) D1**	189 (+/−42)	215 (+/−33)	229 (+/−57)	231 (+/−48)
**Mean tumor vol (mm3) D21**	2165 (+/−370)*	2130 (+/−330)**	1139 (+/−180)* **	1501 (+/−272)
**Relative mean tumor volume D21**	11.5	9.9	5	6.5
**Tumor inhibition rate**	-	13.8	56.7	43.4

* ; **significant differences between tumor volumes (p<0.05).

### Lower Activity of the Combination Doxorubicin/everolimus

The combination of doxorubicin with everolimus had lower therapeutic efficiency than everolimus used alone (p = 0.08) and showed an intermediate additive effect in comparison to doxorubicin (p = 0.1) (Table 1 and Figure 1A). Median tumor burden measured after three weeks of treatment was 1500 mm^3^ (+/−272) in the combination treated group versus 1140 mm^3^ (+/−180) in everolimus-treated rats. The time to achieve the 10-fold initial tumor volume was 17 days in the combination group, vs. 16 days in the doxorubicin treated group. Therefore, the slight tumor growth delay observed in this group was due to everolimus activity, indicating the antagonistic effect of the combination *in vivo*. This lack of synergism between everolimus and doxorubicin was also found *in vitro* in cell proliferation assay. *In vitro* everolimus by itself had no antiproliferative effect on chondrosarcoma and osteosarcoma cell lines (Figure S1) even at the concentration of 1 µM whereas doxorubicin showed a potent antiproliferative effect on both cell lines with an IC 50 of 0.1 µM (Figure S1) These data were not surprising given the mechanism of action of everolimus which is not a cytotoxic agent as opposed to doxorubicin. The addition of everolimus to doxorubicin did not improve the *in vitro* antiproliferative activity of the latter. More studies are ongoing to understand the somewhat antagonistic effect of these two drugs.

### MTOR Inhibition Caused Changes in Tumor Cells Metabolism and Proliferation

**Figure 2:**
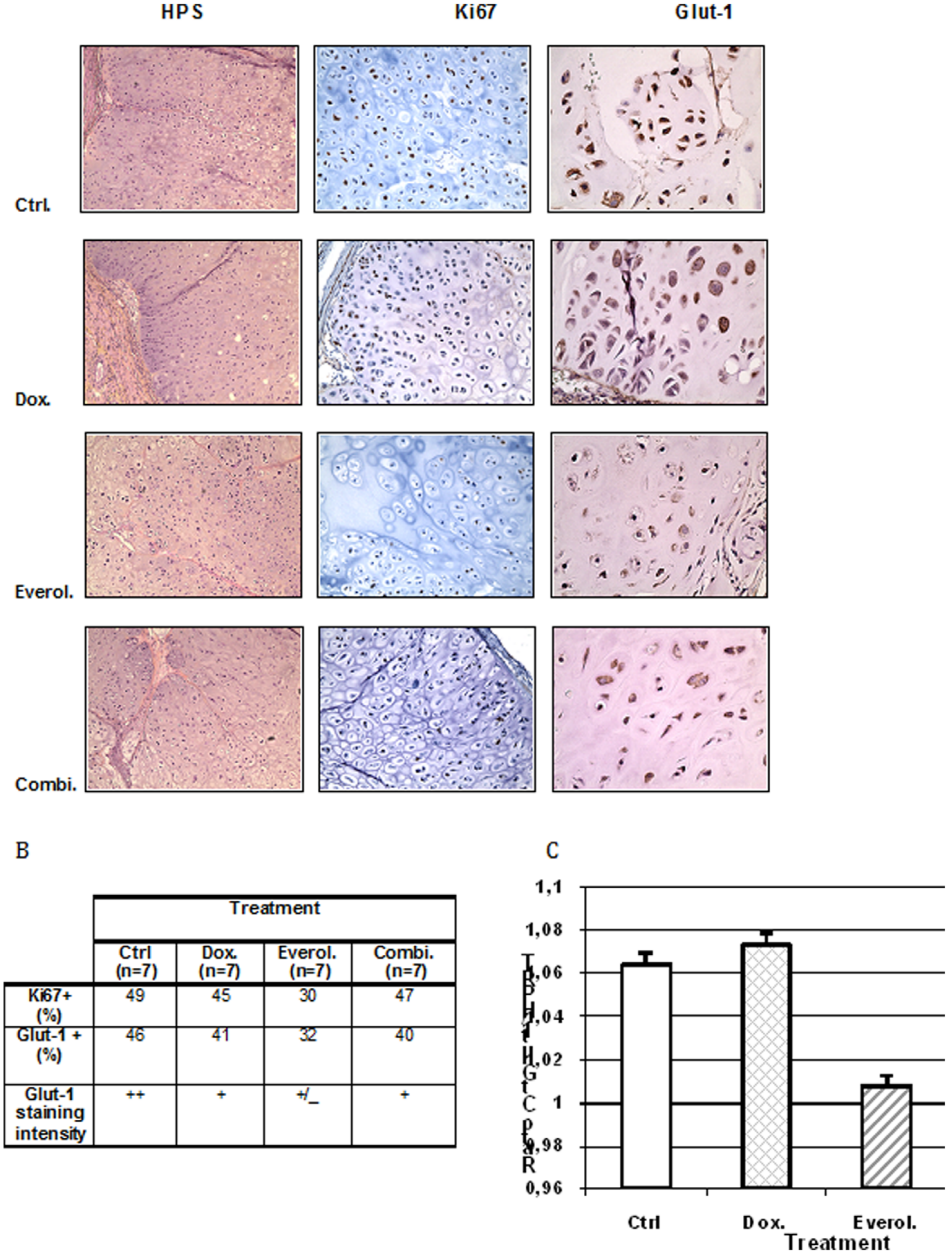
Everolimus inhibits tumor cell proliferation and glucose metabolism. A, Tumor cell proliferative rate (Ki67+ cells) and glucose metabolism were significantly decreased after everolimus treatment. Tumors were harvested, fixed and stained by HPS, anti-Ki67 or anti-Glut-1 antibody to respectively show tumor proliferative fraction and glucose metabolism. B : Summary of Ki67 and Glut-1 expression for each treatment group.Ctrl: control group, Dox: doxorubicin-treated group; Everol: everolimus-treated group; combi: everolimus + doxorubicin combination-treated group. C: histogram showing Glut-1 mRNA expression after each treatment. Glut-1 mRNA induction was unchanged in response to doxorubicin and decreased after everolimus treatment.

After three weeks of treatment, no induction of apoptosis or increase in tumor necrosis was observed histologically in either treated groups (Figure 2A). A reduction of cell proliferation rate was observed in everolimus treated tumors using Ki67 labeling. (Figure 2A and Figure 2B). At the end of the experiment, 30% of tumor cells showed a positive Ki67 staining in the everolimus-treated tumors, 45% in doxorubicin treated tumors and 49% in control group (Figure 2B). The difference in Ki67 positive cells observed between the control or the doxorubicin-treated group and everolimus treated groups were significant (p<0.01 in both cases) whereas only marginal difference seen between the control and doxorubicin treated group was not significant (p = 0.054).

Using immunohistochemistry and RT qPCR, we evaluated the expression of the glucose transporter Glut-1. Interestingly a markedly decreased expression of Glut-1 was observed in the everolimus and combination groups, while a more limited decrease of this marker was observed in the doxorubicin- treated group (Figure 2A and Figure 2B). Glut-1 expression was moderate and observed in 46% of tumor cells in the control group, while it was of low intensity and in 40% of tumor cells in the doxorubicin group (Figure 2). In the everolimus treated tumors, 32% of tumor cells expressed the glucose transporter at a weak level: this percentage was similar in tumors treated with the combination doxorubicin/everolimus. This effect of everolimus on the expression of glucose transporter Glut 1 was also seen at the molecular level. RT qPCR showed a decrease in the expression of GLUT-1 mRNA in the everolimus treated groups whereas no variation in the GLUT-1 mRNA level was found in the doxorubicin treated one. (Figure 2C).

**Figure 3:**
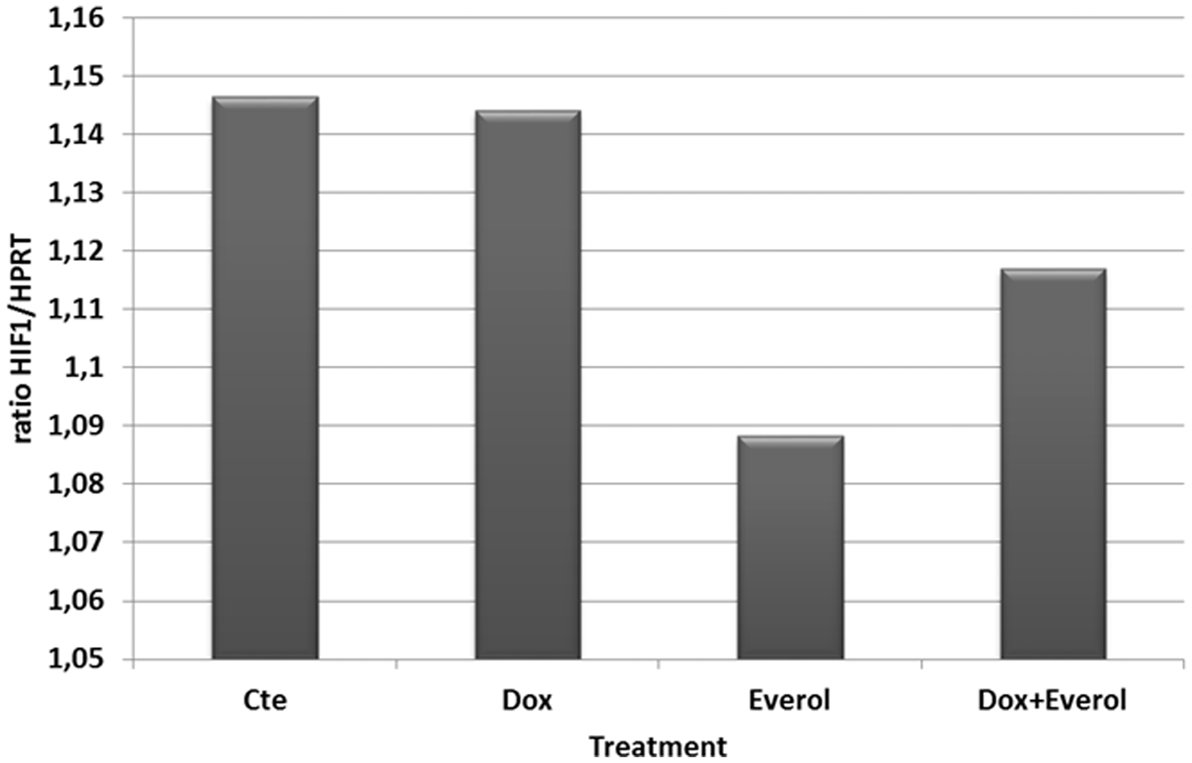
Everolimus did not affect the expression of the hypoxia key regulator HIF1α. RT-qPCR detection of HIF1α mRNA. HIF1α mRNA levels were expressed as a ratio of the corresponding HPRT mRNA level. Results indicate that everolimus induced a slight decrease (P = 0,05) in expression of HIF1α.

The slight decrease in HIF1α expression (Figure 3) suggests that the decreased Glut-1 expression is not due to changes in oxygen levels or tumor hypoxia. The decreased Glut-1 expression seen after treatment by everolimus alone, together with a less important decrease in Glut-1 expression observed in the doxorubicin/everolimus treated group and the absence of changes of Glut-1 expression in the doxorubicin group points to a metabolism inhibitor effect linked to mTOR inhibition (Figure 2 and Figure 3). The correlation seen between Ki67 and Glut 1 staining suggests that everolimus inhibits chondrosarcoma progression mainly by inhibiting cell proliferation and down regulating tumor metabolism.

### Everolimus Blocked mTOR Pathway with no Akt Feedback Loop

**Figure 4:**
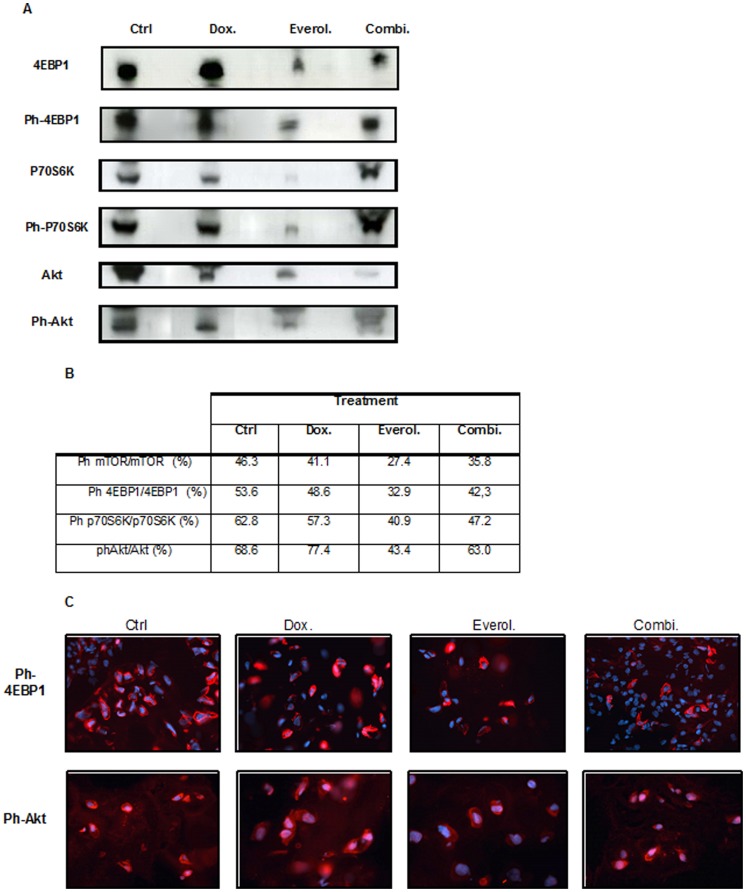
Assessment of mTOR pathway inhibition in everolimus-treated tumors. A, a decrease in the expression of phospho-4EBP1 and phospho-p70S6K1 was observed after treatment with everolimus showing the inhibition of mTOR pathway. Such a decrease was not obtained in the Doxorubicin treated tumors. The expression of ph-Akt was not altered by treatment. B: inhibition of mTOR and its downststream effectors activation after everolimus treatment was confirmed by a decrease of the ratio phosphorylated/total proteins (mTOR 4EBP1, p70S6K1, Akt). C, immunofluorescent staining of ph-4EBP1 and ph-Akt confirmed the Western blot analysis. A strong decrease in ph-4EBP1 staining was seen in tumors from the everolimus treated groups (either as single agent or combination treatment). Neither of the treatments induced changes in the expression of ph-Akt.

Western blot combined with immunohistological analyses showed a strong expression of phospho-Akt, phospho-mTOR, and phospho-p70S6K in the orthotopic chondrosarcoma model (Figure 4A–C), indicating that the mTOR signaling pathway is activated in chondrosarcoma. We evaluated the effects of the different treatments on mTOR pathway targets by immunohistochemical staining and western blotting.

Doxorubicin alone did not decrease mTOR and mTOR effectors activation levels No significant changes in p70S6K1 and 4EBP1 phosphorylation were observed in this group of tumors (Figure 4A–B). The phosphorylated/total protein ratios of mTOR effectors p70S6K1 and 4EBP1 were respectively of 48.6% and 57.3% in doxorubicin treated group versus 53.6% and 62.8% in the control group. In contrast, treatment with everolimus resulted in a significant inhibition (p<0.05) of p70S6K1 and 4EBP1 phosphorylation (ratio of phosphorylated form respectively 40.9% and 32.9%) (Figure 4A–B) confirming the inhibition of downstream signaling of mTOR. Western blot analysis of total proteins from the combination doxorubicin/everolimus treated tumors showed that this treatment inhibits mTOR, p70S6K1 and 4EBP1 phosphorylation but to a lesser level than everolimus alone. Everolimus alone did not led to an increase in Akt phosphorylation in the chondrosarcoma model as seen by western blotting and immunofluorescent stainings (Figure 4); in contrast an increase in Akt phosphorylation could be seen by western blot in the doxorubicin treated group (77.4% of Akt was phosphorylated in these tumors) in comparison to the control one where 68% of Akt was in its activated form in the control group. These data were confirmed by immunofluorescence in tumors receiving doxorubicin alone (Figure 4). In this model and these conditions, everolimus did not activate the feedback TORC2 loop on Akt activation: the feedback was activated in response to doxorubicin and to a lesser extent to the combination doxorubicin/everolimus (Figure 4).

HIF1α is a key element in tumor hypoxia and is overexpressed in chondrosarcoma. This element is partly under the dependance of mTOR signaling. The capacity of everolimus to downregulate HIF1α expression was then tested. RT-PCRq established a slight decrease in HIF1α expression in tumors receiving everolimus as single agent or combined to doxorubicin whereas the chemotherapy alone did not induced changes in HIF1 expression (Figure 3) (from 1.12 to 1.08; P>0.05).

### Adjuvant Everolimus Delays Chondrosarcoma Recurrence

**Figure 5:**
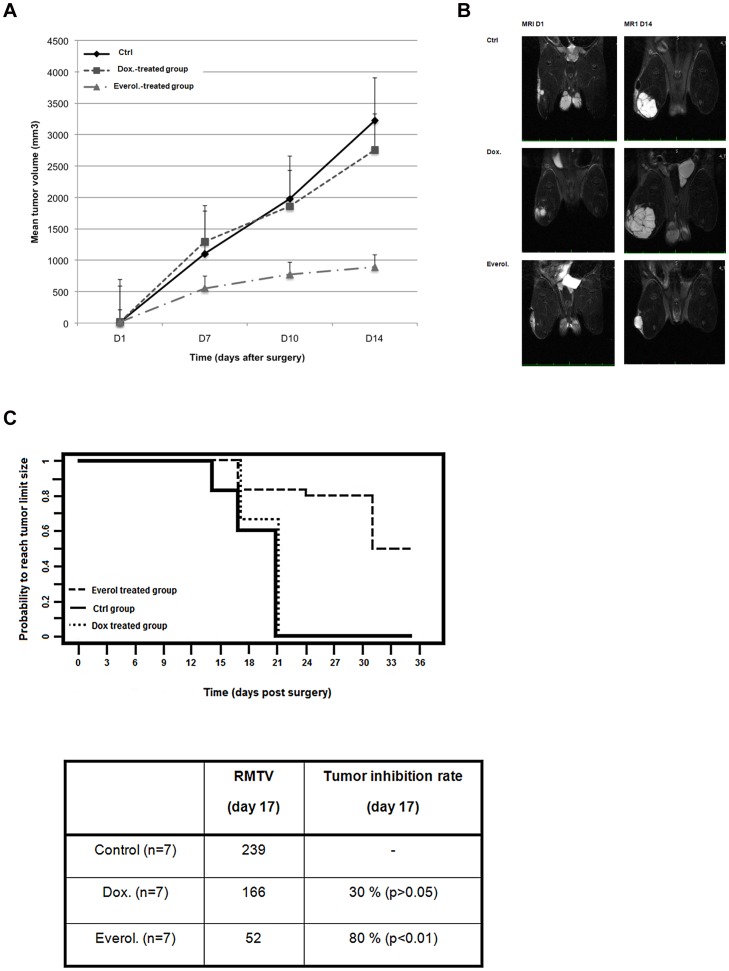
Everolimus as adjuvant treatment delayed chondrosarcoma relapse. A: graph showing mean tumor growth in each group between day 1 and day 14 after surgery. A significant difference in tumor progression was observed between the everolimus treated group and the other groups. B: Longitudinal growth of tumor of one rat from each group monitored by MRI. C: Kaplan Meyer curve and statistical analysis of control and drug-treated groups showing the probability for animals of being event-free (i.e. for tumors to reach 2 cm in diameter). The statistical analyses indicate a significant benefit of everolimus treatment to delay chondrosarcoma progression (P<0.01) over control and doxorubicin. RMTV: relative mean tumor volume as compared to day 1 initiation of treatment.

We explored everolimus in an “adjuvant” setting using the chondrosarcoma model after intralesional curettage: everolimus (1 mg/kg) or doxorubicin (1 mg/kg) treatment was initiated the day after surgery and rats were followed until tumors reached an approximate diameter of 2 cm, at which time the animals were sacrificed (Figure 5). For these conditions, data presented are one experiment representative of the two experiments conducted. Local regrowth was not abolished in everolimus-treated animals but it occurred significantly later in comparison to control and doxorubicin treated animals. At all time points, the mean tumor volume was significantly smaller for everolimus treated animals than in the control and doxorubicin treated groups (Figure S2). At day 14 when all animals were still alive, the mean tumor volume was 3400 mm^3^ (+/−370), 2950 mm^3^ (+/−340) and 900 mm^3^ (+/−300) respectively in the control, doxorubicin and everolimus treated groups (Figure 5A and Figure 5B). In this setting doxorubicin did not cause a delay in tumor regrowth; the difference observed between the control rats and the doxorubicin treated rats was not significant (p = 0.052) while everolimus induced a dramatic slowdown of tumor progression. Progression between day 1 and 17 was significantly higher in control and doxorubicin treated groups than for the animals receiving everolimus (p = 0.004 and p = 0.01 respectively for the comparison of control and everolimus treated groups and for the comparison of doxorubicin and everolimus treated groups, Figure 5A). Using Kaplan-Meier plots, everolimus significantly delayed the time for tumors to reach a 2 cm diameter (P<0.001) (Figure 5B). In the everolimus treated group, 50% of the animals did not reach this critical size 40 days after surgery at which point the animals were sacrificed, whereas in the doxorubicin and control groups all the animals had reached this volume as early as day 18 (Figure 5A and Figure S2). Ki67 and Glut-1 immunohistological analyses showed a high decrease in Ki67+ cells and Glut-1 expression in the everolimus treated tumors in comparison to the control and doxorubicin treated tumors (data not shown).

## Discussion

In this work, we demonstrate the therapeutic role of mTOR inhibition in chondrosarcoma in localized and advanced phase. Everolimus was tested in an orthotopic rat grade II chondrosarcoma model in macroscopic and “adjuvant” phase both reaching the same conclusion. As a single agent, the mTOR inhibitor everolimus did not cause tumor regression but induced a significant inhibition of tumor growth. Both the size and tumor growth rate were smaller in the everolimus treated groups than in other groups, as observed in other tumor models [13], [18], [19], [21], [22].

Doxorubicin was inactive as single agent; when combined with everolimus, an antagonistic effect was actually observed in the combination group compared to the everolimus treated group. When compared to doxorubicin alone, the combination treatment showed however an increased therapeutic efficiency. Although these data are strongly contrasting with those observed in breast cancer models with paclitaxel and prostate cancer with doxorubicin [23], [24], a similar effect was recently reported. In human cervical carcinoma xenograft models the addition of everolimus to doxorubicin showed an antitumor effect that was not significantly different from doxorubicin monotherapy [25].

The mechanisms underlying this lack of synergism between the two drugs are unclear. One of the side effects of doxorubicin treatment is the induction of reactive oxygen species which in turn can activate the Raf/MEK/ERK and PI3K/PTEN/Akt/mTOR pathways [26]–[29]. This activation of the mTOR/Akt pathway induced by doxorubicin is reflected by slight increase in Akt phosphorylation in the doxorubicin treated group of our study. In the case of combined treatment this doxorubicin-induced Akt phosphorylation may not be overcome by everolimus at the concentration used and may counteract the antitumor activity of everolimus, as suggested by the higher expression of phospho Akt of the combination group compared to the everolimus-treated one.

In the chondrosarcoma model the activity of the mTOR pathway in response to the different treatments was monitored by following activation levels of 4EBP1, S6K as potential surrogate markers of tumor response. Measurement of the phosphorylation status of ph-p70S6K1 and ph-4EBP1 in the tumor itself, confirmed that everolimus resulted in a downregulation of mTOR downstream effectors, whereas doxorubicin had no effect on its phosphorylation status. Everolimus exposure alone did not result in the activation of Akt, a phenomenon already reported in other studies [30]–[32]. It is known that mTOR inhibitor– can induce a feedback activation of Akt thus contributing to a lesser therapeutic efficiency [32]. This was not observed here with everolimus alone.

The data obtained in these experiments indicate that everolimus may affect cell proliferation and metabolism as shown by the down regulation of Ki67 and Glut1 immunostaining. Such an antiproliferative effect has already been reported [12]. The significantly decreased GLUT1 expression observed in the everolimus treated groups appears to be the result of mTOR inhibition and is a consequence of the cross-talk of mTOR downstream effectors with metabolic and hypoxic pathways [33]. Inhibition of mTOR signaling may have direct effect on cell proliferation and also an indirect inhibitor effect on glucose metabolism through the inhibition of HIF1α which expression is dependent upon mTOR [6], [33]. The decrease in HIF1α expression seen by immunofluorescence and in the levels of HIF1 α transcript seen by RT-qPCR in tumors of the everolimus treated groups support this bifunctional action of everolimus.

Importantly, the present study also investigated the effects of everolimus on residual disease after intralesional curettage in the rat model of chondrosarcoma. In contrast to doxorubicin which was unable to inhibit chondrosarcoma regrowth, everolimus treatment significantly delayed local recurrence in the treated group but did not prevent it after intralesional curettage. The preclinical model used in this study reproduces thus clinical situations in large chondrosarcoma. This suggests that everolimus could be worth exploring as adjuvant treatment at least in patients with grade 2 or higher chondrosarcoma. Whether everolimus would be able to show the same antitumor activity in all chondrosarcoma subtypes will be tested in a prospective randomized trial scheduled to be activated in 2012 in the French Sarcoma Group.

Although everolimus as monotherapy showed a strong antitumor effect and did not induce an increase in phosphorilated Akt in our chondrosarcoma model one cannot put aside the possibility that resistance could emerge in response to long term mTORC1 inhibition. It is known that blockade of mTOR signaling by rapalogs leads to loss of feedback inhibition on Akt [34]. That could potentially result in increased cell survival and resistance to cancer therapy [35]. To prevent such resistance mechanism and additionally improve everolimus therapeutic efficiency everolimus-based combination therapy could be envisionned. Such dual targeted approaches targeting mTOR and Akt [36], or mTOR and PI3K have proven to be pertinent in preclinical models [34] and one (targeting mTOR and IGFR-1) has reached the clinical phase in patients with advanced sarcomas and other solid tumors [35].

Another possible combination could be to add a bone remodelling agent to everolimus.

Indeed, the combination of zoledronate to everolimus was effective in inhibiting tumor progression and in protecting bone in murine osteosarcoma model [37]. The latter effect being the result of zoledronate rather than the one of everolimus. Like osteosarcoma, chondrosarcoma is characterized by a tumor-induced osteolysis; moreover, zoledronate has already proven to be an efficient agent in the same chondrosarcoma model [38]. Thus it seems pertinent to hypothesize that the combination of everolimus to zoledronate could be efficient in this tumor. Such combined therapies are worth exploring in preclinical settings.

In conclusion, the present results show that everolimus would be an effective antitumor agent in chondrosarcoma. Besides, the inhibition of tumor regrowth following surgery suggests that everolimus could be used as adjuvant long-term therapy in chondrosarcoma patients following surgery. These results open the way to new therapeutic approaches and led to a prospective phase II clinical trial initiatied in the French Sarcoma Group.

## Supporting Information

Figure S1 **Effects of everolimus and doxorubicin on sarcoma cell proliferation** ***in vitro*** **.** Chondrosarcoma (A) and osteosarcoma (B) cells were incubated with increasing concentration of everolimus and doxorubicin. Growth inhibition was analyzed from T0 to T0+96 hrs, using the cell titer glo assay. Absorbance values were normalized to 100% using the values from untreated cells. Results are the mean ± SD of three independent experiments.
(TIF)

Figure S2 **Individual progression of tumor volume after intralesional curetage and corresponding treatment.** All tumors from the control and doxorubicin treated groups reached the limit size of 2 cm in less than 20 days after curettage. A slower progression was obtained Under Everolimus treatment.
(TIF)
