# Supplementary figures and images for: Inhibition of Chondrosarcoma Growth by mTOR Inhibitor in an In Vivo Syngeneic Rat Model
Source: PLoS One. 2012 Jun 27;7(6):e32458. doi: 10.1371/journal.pone.0032458 (PMC3384598; doi:10.1371/journal.pone.0032458)

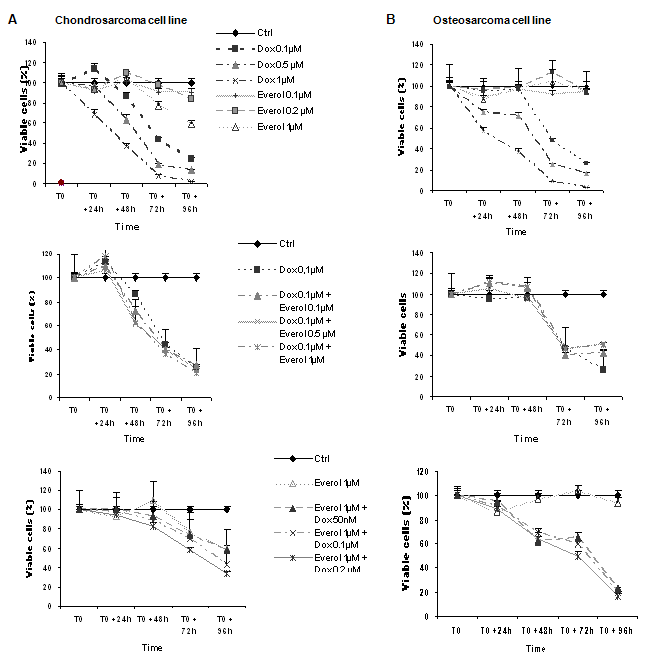

Supplement: Figure S1 — Effects of everolimus and doxorubicin on sarcoma cell proliferation in vitro . Chondrosarcoma (A) and osteosarcoma (B) cells were incubated with increasing concentration of everolimus and doxorubicin. Growth inhibition was analyzed from T0 to T0+96 hrs, using the cell titer glo assay. Absorbance values were normalized to 100% using the values from untreated cells. Results are the mean ± SD of three independent experiments. (TIF) [file pone.0032458.s001.tif]

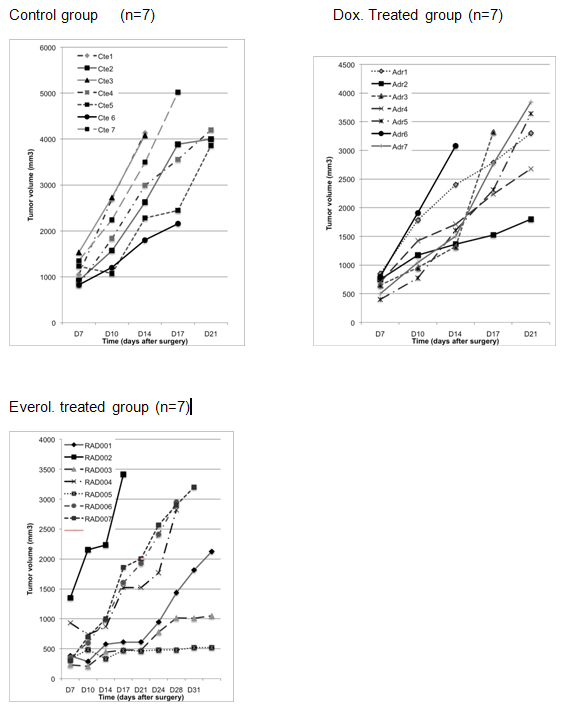

Supplement: Figure S2 — Individual progression of tumor volume after intralesional curetage and corresponding treatment. All tumors from the control and doxorubicin treated groups reached the limit size of 2 cm in less than 20 days after curettage. A slower progression was obtained Under Everolimus treatment. (TIF) [file pone.0032458.s002.tif]
